# Relationship between Metabolic Syndrome and Ocular Microcirculation Shown by Laser Speckle Flowgraphy in a Hospital Setting Devoted to Sleep Apnea Syndrome Diagnostics

**DOI:** 10.1155/2017/3141678

**Published:** 2017-09-05

**Authors:** Tomoaki Shiba, Mao Takahashi, Tadashi Matsumoto, Yuichi Hori

**Affiliations:** ^1^Department of Ophthalmology, Toho University Omori Medical Center, Tokyo, Japan; ^2^Cardiovascular Center, Toho University Sakura Medical Center, Chiba, Japan

## Abstract

**Purpose:**

To clarify whether the incidence of metabolic syndrome (MetS) and the overlap of MetS components are affecting the ocular circulation shown by laser speckle flowgraphy (LSFG).

**Materials and Methods:**

We studied 76 consistent patients. Blowout score (BOS) and blowout time (BOT), which are the pulse waveform analysis parameters, and mean blur rate (MBR) using LSFG in the optic nerve head (ONH) and choroid were evaluated. Throughout, the ONH was separated out from the vessels and tissue for analysis and MBRs in the ONH were divided into four sections (superior, temporal, inferior, and nasal).

**Results:**

Thirty-two patients were diagnosed having Mets. MBR-Tissue (*P* = 0.003), MBR-All (*P* = 0.01), MBR-Choroid (*P* = 0.04), and BOS-Choroid (*P* = 0.03) were significantly lower in patients with MetS than in the patients without MetS. Multiple-regression analysis revealed the temporal side of MBR-Tissue and BOS-Choroid which were identified as factors contributing independently to the overlap of the MetS components. Multiple-regression analysis also revealed that the MetS components were identified to be factors independently contributing to the BOS-Choroid and temporal side of MBR-Tissue.

**Conclusion:**

Our study clarified that the incidence of MetS and the overlap of the MetS components are significantly affecting the ONH and choroidal microcirculation.

## 1. Introduction

The metabolic syndrome (MetS) refers to a cluster of metabolic abnormalities, including obesity, hyperglycemia, dyslipidemia, and hypertension. MetS increases the risk for diabetes mellitus and cardiovascular disease and then increases morbidity and mortality [1–4]. Numerous researchers reported that subjects with MetS had significantly greater carotid intima-media thickness (IMT) compared with subjects without MetS [5–10] and the IMT increases with each additional component of MetS [11]. The reports of the relationship between MetS and cardiac function have revealed that MetS were independently associated with left ventricular diastolic dysfunction [12–15].

Laser speckle flowgraphy (LSFG) is a safe and quantitative devise for evaluating of ocular circulation [16, 17]. This is based on a change in patterns of the speckles of the laser light reflected from a retina and choroid [18, 19]. LSFG depends on the red blood cells in the optic nerve head (ONH), choroid, and retina; the mean blur rate (MBR) is a unique index of blood flow of LSFG [20, 21]. The LSFG-NAVI™ (Softcare Co., Fukuoka, Japan) was approved in 2008 as a medical apparatus by Japan's Pharmaceuticals and Medical Devices Agency and in 2016 by the U.S. Food and Drug Administration (FDA).

Variations in the MBR have pulse wave patterns that are synchronized with the cardiac cycle. We reported that the blowout time (BOT) and blowout score (BOS), factors obtained from a pulse waveform analysis, were significantly correlated with arteriosclerosis and left ventricular diastolic status [22–24]. We hypothesized that the cluster of components of MetS may affect the ocular circulation shown by LSFG. The purpose of the present study was thus to clarify whether the incidence of MetS and the overlap of MetS components are affecting the ocular circulation in the ONH and choroid shown by the LSFG, while comparing them with IMT and left ventricular diastolic function.

## 2. Materials and Methods

### 2.1. Patients

The design of the current study was cross-sectional comparative study.

The institutional review board of Toho University Sakura Medical Center approved the present study (numbers 2011-009 and 2010-012). All participants provided informed consent according to the Declaration of Helsinki. We studied 76 consistent patients who had undergone polysomnography (a test for sleep apnea) and were able to detect the MetS components accurately, at the Department of Cardiovascular Center of Toho University Sakura Medical Center between January 18, 2010, and June 12, 2013. Patients were excluded if they had atrial fibrillation, glaucoma, uveitis, optic neuropathy, vitreous or retinal disease, or retinal or choroidal vascular disease or if they had undergone a previous intraocular surgery. All patients were evaluated while they were hospitalized.

### 2.2. Measurements of Mean IMT

We have described the precise method of measurements of mean IMT in our previous report [25]. Briefly, high-resolution ultrasonographic imaging of the carotid artery using the B-scan mode was performed using the EUB-8500 ultrasound system (Hitachi Co. Ltd., Tokyo, Japan) with the probe frequency set to 7.5 MHz. Imaging was performed with the patients in a supine position with their heads turned slightly away from the sonographer. The procedure involved scanning the near and far walls of the carotid artery every 1 centimeter proximal to the carotid bulb in the longitudinal view. The mean IMT was defined as the average of the maximal IMT 1 centimeter proximal and 1 centimeter distal to the carotid bulb [26, 27]. The mean IMT of the thickened side of the carotid artery was used for data analyses.

### 2.3. Measurements of Left Ventricular Diastolic Function

We have described the precise method of measurements of left ventricular diastolic function in our previous report [25]. Briefly, left ventricular diastolic function was assessed according to the recent consensus guidelines [28, 29] on diastolic function evaluation measuring mitral inflow velocities (E-wave) using pulse wave Doppler in the apical four-chamber view. The pulse wave tissue Doppler velocities were acquired at end-expiration, in the apical four-chamber view, with the sample positioned at the lateral mitral annulus, measuring early diastolic (e' velocity) and calculating the E/e' ratio. The E/e' ratio has been reported to be the single best predictor of the left ventricle diastolic filling pressure [30]. Echocardiography was performed using a commercially available instrument (Vivid 7, GE Healthcare, Japan).

### 2.4. Laboratory Measurements and Systemic Parameters

The following values were measured: fasting blood sugar (FBS: mg/dl), total cholesterol (mg/dl), triglycerides (mg/dl), high-density lipoprotein cholesterol (HDL-C: mg/dl), low-density lipoprotein cholesterol (LDL-C: mg/dl), homeostasis model assessment of insulin resistance (HOMA-IR), hematocrit (%), and creatinine (mg/dl) obtained from fasting morning blood samples. HOMA-IR = fasting insulin (IU/ml) × fasting blood glucose (mmol/l)/22.5 [31]. The body mass index (BMI: kg/m^2^), systolic blood pressure (SBP: mmHg), diastolic blood pressure (DBP: mmHg), and heart rate (beats per min, bpm) were evaluated.

### 2.5. Diagnosis of MetS

The definition by the Japanese Committee to Evaluate Diagnostic Standards for Metabolic Syndrome was used for the diagnosis of MetS [32, 33]. This definition is based on abdominal obesity (waist ≥ 85 cm for men and ≥90 cm for women) plus two or more components of metabolic risk factors, (1) hypertension: SBP ≥ 130 mmHg or DBP ≥ 85 mmHg or those who had been treated for hypertension, (2) dyslipidemia: HDL-C < 40 mg/dl or triglycerides ≥ 150 mg/dl or a history of previous treatment for dyslipidemia, and (3) high glucose: FBS ≥ 110 mg/dl or a history of previous treatment for diabetes.

### 2.6. LSFG Measurements

**Figure 1:**
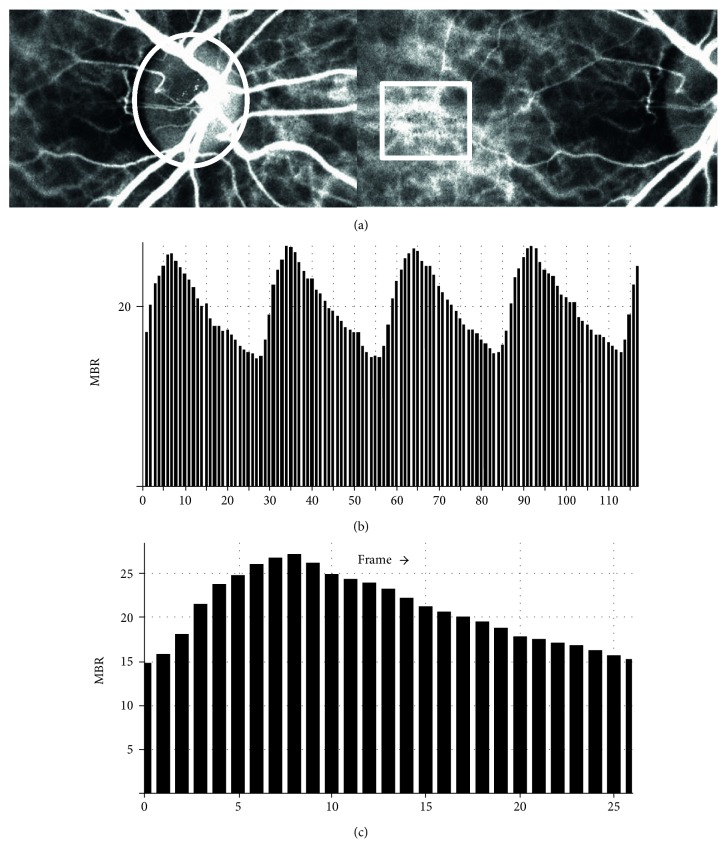
The method for analyzing the mean blur rate (MBR) and pulse waveform in the optic nerve head (ONH) and choroid circulation using LSFG. (a) The gray-scale map of the total measurement area. The circle and rectangle designates the area of the ONH and center placed at the foveal area avoiding the retinal vessel measured. (b) The pulse waves show changes in the MBR, which is tuned to the cardiac cycle for 4 seconds. The total number of frames is 118. (c) Normalization of one pulse. MBRs are made on this screen.

The details of the determination of the LSFG measurements from fundus images were as described [21, 34]. We have described the methods of average MBR, BOT, and BOS which are items of pulse waveform in our previous report [35]. Briefly, for the evaluation of the ONH and choroidal blood flow, a circle was set surrounding the ONH, and the center of a rectangle was placed at the foveal area avoiding the retinal vessel (Figure 1, upper panel).

**Figure 2:**
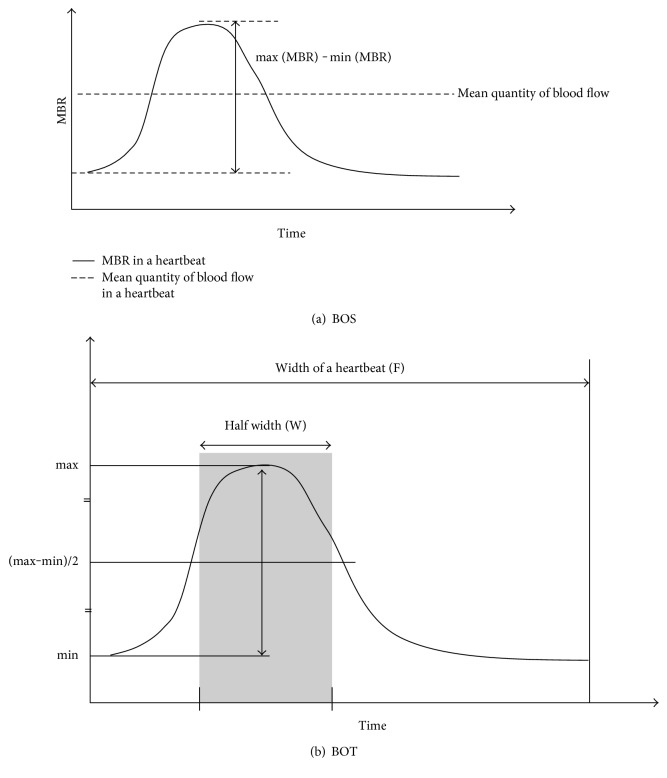
Schematic explanation of the blowout score (BOS) and blowout time (BOT) obtained from pulse waveform analysis.

First, 118 MBR images (118 frames) were recorded from the circle and the rectangle area within a 4 sec period tuned to the cardiac cycle. On the analysis screen, the pulse wave of the changing MBR values which corresponded to each cardiac cycle was obtained (Figure 1(b)). The analysis of the screen which is normalized to one pulse is then displayed (Figure 1(c)), and the analysis of the pulse waveform and average MBR is made on this screen. In the present study, the LSFG used the mean MBR as an indicator of blood flow. The BOS and BOT were also calculated.  Figure 2 shows the schematic explanations of the BOS (Figure 2(a)) and BOT (Figure 2(b)) obtained from waveform analysis. And the BOS and BOT values were determined by the following formulae [21]:

(1) $$\begin{array}{c} BOS=\frac{\left(2-\left(\max MBR-\min MBR\right)/mean\;quantity\;of\;blood\;flow\right)}{2\times 100},\\ BOT=100\times \frac{W}{F}. \end{array}$$

**Figure 3:**
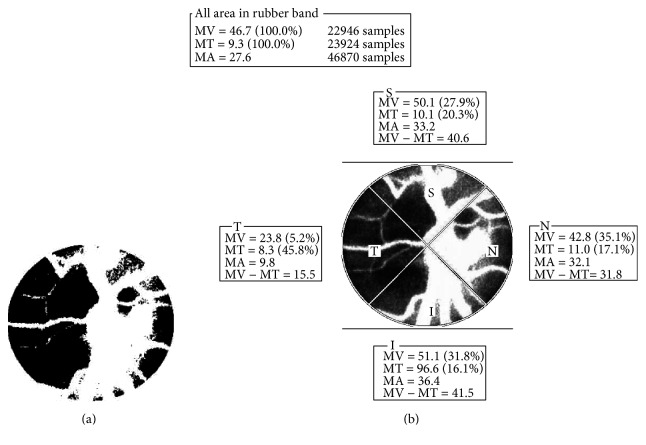
The software segments out the retinal vessels using the automated definitive threshold throughout the ONH, within the ONH vessel (shown in white) and within the ONH tissue (shown in black) (a). Next, the software divided into 4 sections (superior, temporal, inferior and nasal) (b) and analyzes the mean blur rate in the each section. T = temporal; N = nasal; S = superior; I = inferior.

We calculated parameters using LSFG Analyzer software (v.3.0.47, Softcare Co. Ltd., Fukuoka, Japan). Next, the software separated out the vessels using the automated definitive threshold (Figure 3(a)) and then analyzed the means of the MBR, BOS, and BOT in the ONH tissue (Tissue), in the vessels of the ONH (Vessel), and throughout the ONH (All). Finally, MBRs in the ONH were divided into four sections (superior, temporal, inferior, and nasal: Figure 3(b)). All patients were measured in a seated position, and pupils were dilated with 0.5% tropicamide eye drops. Only the data from the right eye were used for the analysis.

### 2.7. Measurements of Other Ocular Parameters

The following parameters of right eyes were measured: spherical refraction (diopters (D)) assessed with the TONOREF 2™ system (NIDEK Co., Aichi, Japan), intraocular pressure (IOP, mmHg) measured by applanation tonometry, and ocular perfusion pressure (OPP, mmHg). The OPP was defined as (2/3 mean arterial blood pressure) − IOP.

### 2.8. Statistical Analysis

Data are presented as the means ± standard deviations for the continuous variables.

Unpaired *t*-test, Yates 2 × 2 chi-square test, 2 × 2 chi-square test, and Fisher's exact probability were used for comparison of clinical and ocular variables between patients with MetS and those without MetS. Single-regression analyses were used to determine the relationship between the overlap of the MetS components and all of the LSFG parameters. Multiple-regression analyses were used to determine the independent factors for MetS components and the LSFG parameters which were significantly correlated with the MetS components. *P* values < 0.05 were considered significant. The StatView v 5.0 program (SAS Institute, Cary, NC) was used for statistical analyses.

## 3. Results

**Figure 4:**
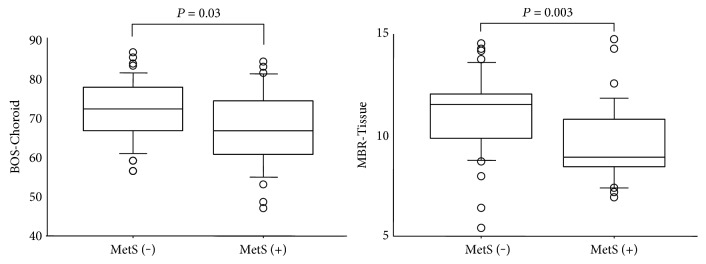
The value of blowout score- (BOS-) Choroid and mean blur rate- (MBR-) Tissue in the optic nerve head in the patients with and without metabolic syndrome. Statistical differences between groups were calculated by unpaired *t*-test.

Tables 1 and 2 show the results of a comparison of clinical and LSFG parameters in patients with and without MetS. Thirty-two patients were diagnosed having MetS. The ratio of men, BMI, E/e' ratio, FBS, triglyceride, HOMA-IR, and frequency of hypertension and diabetes were significantly higher in the patients with MetS than in the patients without MetS. MBR-Tissue, MBR-All, MBR-Choroid, and BOS-Choroid were significantly lower in patients with MetS than in the patients without MetS (MBR-Tissue MetS (+) versus MetS (−): 9.7 ± 1.9 versus 11.0 ± 2.0, *P* = 0.003; MBR-All: 16.4 ± 4.0 versus 18.7 ± 3.6, *P* = 0.01; MBR-Choroid: 5.4 ± 1.4 versus 6.3 ± 2.0, *P* = 0.04; and BOS-Choroid: 67.4 ± 9.7 versus 71.8 ± 7.6, *P* = 0.03).  Figure 4() represents comparison of MBR-Tissue and BOS-Choroid between patients with MetS and those without MetS.  Table 3 shows the results of the single-regression analysis between MetS components and MBR, BOS, and BOT in the section of Tissue, Vessel, All, and Choroid. MBR-Tissue (*r* = −0.35, *P* = 0.002), MBR-All (*r* = −0.32, *P* = 0.005), MBR-Choroid (*r* = −0.27, *P* = 0.03), and BOS-Choroid (*r* = −0.27, *P* = 0.02) were significantly negatively correlated with the MetS components. Next, we divided the MBR-Tissue which showed the strongest correlations with the MetS components into 4 sections (superior, temporal, inferior, and nasal), and Table 4 shows the results of the single-regression analysis between MetS components and these parameters. MBR-Tissues—superior (*r* = −0.29, *P* = 0.01), inferior (*r* = −0.31, *P* = 0.007), and temporal (*r* = −0.40, *P* = 0.0004)—were significantly negatively correlated with the overlap of MetS components.  Table 5 shows the results of the multiple-regression analysis for factors contributing independently to MetS components; the explanatory variables were the parameters that were significantly different in patients with and without MetS and mean IMT. The ratio of men HOMA-IR, temporal side of MBR-Tissue, and BOS-Choroid were identified as factors contributing independently to the overlap of the components (men = 1, women = 0: standard regression coefficient = 0.45, *t* value = 4.79, *P* < 0.0001; HOMA-IR: standard regression coefficient = 0.31, *t* value = 3.03, *P* = 0.003; MBR-Tissue (temporal): standard regression coefficient = −0.24, *t* value = −2.63, *P* = 0.01; and BOS-Choroid: standard regression coefficient = −0.23, *t* value = −2.52, *P* = 0.01).

The single-regression and multiple-regression analyses of factors independently contributing to the BOS-Choroid and MBR-Tissue (temporal) are shown in Tables 6 and 7. The MBR-Choroid was significantly correlated with age (*r* = −0.45, *P* < 0.0001), heart rate (*r* = 0.28, *P* = 0.01), hematocrit (*r* = 0.30, *P* = 0.009), and MetS components (*r* = −0.27, *P* = 0.02). And age (standard regression coefficient = −0.41, *t* value = −3.47, *P* = 0.0009), heart rate (standard regression coefficient = 0.44, *t* value = 4.89, *P* < 0.0001), and MetS components (standard regression coefficient = −0.31, *t* value = −3.41, *P* = 0.001) were identified to be factors independently contributing to the BOS-Choroid. MBR-Tissue (temporal) was significantly correlated with heart rate (*r* = −0.25, *P* = 0.03), mean IMT (*r* = −0.28, *P* = 0.01), and MetS components (*r* = −0.40, *P* = 0.0004). And mean IMT (standard regression coefficient = −0.22, *t* value = −2.03, *P* = 0.046) and MetS components (standard regression coefficient = −0.31, *t* value = −2.84, *P* = 0.006) were identified to be factors independently contributing to the MBR-Tissue (temporal).

## 4. Discussion

In this study, and definition by the Japanese Committee to Evaluate Diagnostic Standards for Metabolic Syndrome was used for the diagnosis of the association between MetS [33, 34] and ocular circulation in the ONH and choroid shown by LSFG.

There were several reported relationships between the MetS and ocular findings. It has been reported that subjects with more MetS components had higher IOP [36]. Another study reported that the overlap of MetS components were predictors of progression to late age-related macular degeneration [37]. As an important evidence, it was clarified that MetS components, hypertension, and impaired FBS were contributing to an increasing risk of normal-tension and open-angle glaucoma [38, 39]. However, to the best of our knowledge, it was still unknown whether the incidence of MetS and the overlap of the MetS components affect the ocular circulation, including ONH and choroid. The purpose of the present study was thus to elucidate whether the overlap of the MetS components is affecting the ocular circulation obtained from LSFG.

In the analysis for patient's characteristics of our study, age was not significant different between patients with MetS and those without MetS. On the other hand, the ratio of men in patients with MetS was significantly higher than the ratio of men in patients without MetS. The E/e' ratio in patients with MetS was significantly higher than that in patients without MetS. On the other hand, IOP was not significantly different between patients with MetS and those without MetS.

Our analysis of the relationship between LSFG measurements and MetS revealed that MBR-Tissue, MBR-All, MBR-Choroid, and BOS-Choroid in patients with MetS were significantly lower than those in patients without MetS. In addition, the single-regression analysis showed that MBR-Tissue, MBR-All, MBR-Choroid, and BOS-Choroid were significantly correlated with the overlap of the MetS components. Especially, MBR-Tissue has the strongest correlations with the components of MetS. It was reported that MBR-Tissue in the ONH was strongly correlated with capillary blood flow obtained from hydrogen gas clearance technique [40]. Therefore, the overlap of the MetS components may lead to a decrease of the capillary blood flow in the ONH. The BOS represent the changing of the MBR during the cardiac cycle; thus, our results show changing of the MBR in the choroidal area which is wider in parallel with the cumulation of the MetS components. Previous few researchers reported that patients with MetS were more likely to have microvascular changes, for example, arteriovenous nicking, focal arteriolar narrowing, enhanced arteriolar wall reflex, retinopathy, and smaller arteriolar diameter [41–43]. Thus, we think that further study will be needed to clarify relationships between hemodynamic change in the ONH and choroid and the morphological microvascular change due to MetS.

Next, we divided MBR-Tissue in the ONH into four sections (superior, temporal, inferior, and nasal). In the results, the temporal side of MBR-Tissue has strongest correlations with the overlap of the MetS components.

The multiple-regression analysis showed that the ratio of men, HOMA-IR, temporal side of MBR-Tissue, and BOS-Choroid were identified as factors contributing independently to the overlap of the MetS components, comparing them with IMT and left ventricular diastolic function. It is well known that there is a strong correlation between insulin resistance and MetS [44, 45]. It was suggested that the temporal side of MBR-Tissue and BOS-Choroid is more strongly correlated with MtS components than mean IMT and E/e' ratio which are reported as contributing factors for MetS.

Our single-regression and multiple-regression analyses revealed that the age, heart rate, and overlap of MetS components were identified as factors contributing independently to the BOS-Choroid. In addition, mean IMT and the overlap of the MetS components were also identified as factors contributing independently to the temporal side of MBR-Tissue. It was suggested that the overlap of MetS components is one of the important factors for defining the ocular circulation in the ONH and choroid.

Previous other studies have confirmed that the tissue area of MBR and pulse waveforms in the ONH were well associated with sensitivity of the visual field in patients with normal-tension glaucoma [46, 47]. From the point of view of ocular circulation, our finding may be important clues to understand the mechanism of the progression of open-angle glaucoma due to the incidence of MetS and the overlap of the MetS components. In addition, a previous population-based study reported that there was no evidence of an association between the MetS and retinopathy independent of diabetes status [48]. Conversely, our study clarified that the incidence of MetS and the overlap of the MetS components are significantly affecting the ONH and choroidal circulation, from the stage of the absence of retinopathy.

There are some major limitations in this study. First, our subjects of the current study included some patients with sleep apnea syndrome. The apnea-hypopnea index which is the major outcome of polysomnography did not correlate with BOS and MBR in each section of ONH and choroid (data not shown). Thus, we considered that the influence that sleep apnea had on the results of the current study was low. However, there may be a slight selection bias in this study. Second, we did not evaluate the visual field and peripapillary retinal nerve layer thickness using optical coherence tomography. Thus, a more detailed further evaluation will be needed to clarify the relationships between ocular circulation and open-angle glaucoma in patients with MetS. Third, the use of calcium channel blocker and angiotensin-converting enzyme inhibitor or angiotensin 2 receptor blocker was significantly higher in the MetS group. Because the current study was a cross-sectional study, the effect of these drugs was not able to be evaluated. A prospective study is needed to determine whether treatment of MetS components prevents decreases in the temporal side of MBR-Tissue and BOS-Choroid. Finally, the current results were obtained using relatively small sample sets. Clearly, further careful validation studies with more samples are needed to evaluate the effects of MetS on the ocular circulation as primary endpoints.

In conclusion, our study clarified that the incidence of MetS and the overlap of the MetS components are significantly affecting to the ONH and choroidal circulation obtained from LSFG.

## Figures and Tables

**Table 1:** Comparison of clinical variables between in patients with and without metabolic syndrome.

	MetS (+) *n* = 32	MetS (−) *n* = 44	*P*
Age (yrs)	61.6 ± 11.0	62.3 ± 11.6	0.79^†^
Men : women	28 : 4	27 : 17	0.02^‡^
BMI (kg/m^2^)	27.0 ± 3.6	24.1 ± 3.8	0.001^†^
SBP (mmHg)	133.0 ± 18.4	128.1 ± 17.1	0.24^†^
DBP (mmHg)	75.5 ± 11.0	70.5 ± 11.1	0.06^†^
Spherical refraction (D)	−0.93 ± 2.71	−0.25 ± 2.33	0.25^†^
IOP (right: mmHg)	12.9 ± 2.9	12.4 ± 3.2	0.45^†^
OPP (mmHg)	50.2 ± 8.4	47.4 ± 8.2	0.16^†^
Heart rate (bpm)	69.8 ± 9.7	66.3 ± 9.2	0.12^†^
Mean IMT (mm)	1.18 ± 1.44	0.88 ± 0.21	0.18^†^
e' velocity (cm/s)	6.1 ± 1.5	6.6 ± 2.1	0.20^†^
E/e' ratio	13.0 ± 4.7	11.0 ± 2.9	0.03^†^
Hypertension (%)	26 (81.3)	18 (40.9)	0.001^‡^
Diabetes (%)	16 (50.0)	4 (9.1)	0.0001^‡^
FBS (mg/dl)	120.8 ± 29.8	94.9 ± 11.2	<0.0001^†^
Total cholesterol (mg/dl)	191.5 ± 27.1	198.5 ± 29.6	0.30^†^
Triglyceride (mg/dl)	163.3 ± 69.5	123.7 ± 51.4	0.006^†^
HDL-C (mg/dl)	49.8 ± 19.9	57.0 ± 14.2	0.07^†^
LDL-C (mg/dl)	114.2 ± 25.2	115.8 ± 24.6	0.78^†^
HOMA-IR	2.8 ± 2.6	1.5 ± 0.9	0.002^†^
Hematocrit (%)	42.3 ± 4.3	40.8 ± 3.9	0.13^†^
Creatinine (mg/dl)	0.84 ± 0.14	0.83 ± 0.17	0.81^†^

Mean ± standard deviation; ^†^unpaired *t*-test; ^‡^Yates 2 × 2 chi-squared test. MetS: metabolic syndrome; BMI: body mass index; SBP: systolic blood pressure; DBP: diastolic blood pressure; D: diopter; IOP: intraocular pressure; OPP: ocular perfusion pressure; IMT: intima-media thickness; FBS: fasting blood sugar; HDL-C: high-density lipoprotein cholesterol; LDL-C: low-density lipoprotein cholesterol; HOMA-IR: homeostasis model assessment of insulin resistance.

**Table 2:** Comparison of items of ocular circulation between patients with and without metabolic syndrome.

	MetS (+) *n* = 32	MetS (−) *n* = 44	*P*
MBR-Tissue	9.7 ± 1.9	11.0 ± 2.0	0.003
MBR-Vessel	32.5 ± 7.6	35.0 ± 7.3	0.15
MBR-All	16.4 ± 4.0	18.7 ± 3.6	0.01
MBR-Choroid	5.4 ± 1.4	6.3 ± 2.0	0.04
BOS-Tissue	73.8 ± 8.2	74.5 ± 6.7	0.69
BOS-Vessel	77.8 ± 6.5	78.6 ± 5.3	0.54
BOS-All	76.2 ± 7.2	77.1 ± 5.7	0.55
BOS-Choroid	67.4 ± 9.7	71.8 ± 7.6	0.03
BOT-Tissue	46.1 ± 5.1	46.5 ± 4.3	0.77
BOT-Vessel	50.2 ± 4.0	51.1 ± 4.1	0.35
BOT-All	48.2 ± 4.0	47.4 ± 8.2	0.29
BOT-Choroid	43.8 ± 4.6	45.2 ± 3.9	0.17

Mean ± standard deviation; unpaired *t*-test. MetS: metabolic syndrome; MBR: mean blur rate; BOS: blowout score; BOT: blowout time.

**Table 3:** Correlation coefficients of a single-regression analysis between items of ocular circulation and components of metabolic syndrome.

Explanatory variables	MBR		BOS		BOT	
	*r*	*P*	*r*	*P*	*r*	*P*
Tissue	−0.35	0.002	−0.03	0.78	−0.001	0.99
Vessel	−0.20	0.08	−0.04	0.76	−0.11	0.37
All	−0.32	0.005	−0.04	0.74	−0.12	0.31
Choroid	−0.24	0.03	−0.27	0.02	−0.14	0.24

Objective variables: components of metabolic syndrome (0, 1, 2, and 3). *n* = 76. MBR: mean blur rate; BOS: blowout score; BOT: blowout time.

**Table 4:** Correlation coefficients of a single-regression analysis between 4 quadrants (superior, nasal, inferior, and temporal) of mean blur rate- (MBR-) Tissue and components of metabolic syndrome.

Explanatory variables	*r*	*P*
MBR-Tissue (superior)	−0.29	0.01
MBR-Tissue (nasal)	−0.20	0.08
MBR-Tissue (inferior)	−0.31	0.007
MBR-Tissue (temporal)	−0.40	0.0004

Objective variables: components of metabolic syndrome (0, 1, 2, and 3). *n* = 76.

**Table 5:** Results of a multiple-regression analysis for factors independently contributing to components of metabolic syndrome.

Explanatory variables	Standard regression	*t* value	*P*
Men = 1, women = 0	0.45	4.79	<0.0001
HOMA-IR	0.31	3.03	0.003
MBR-Tissue (temporal)	−0.24	−2.63	0.01
BOS-Choroid	−0.23	−2.52	0.01
E/e' ratio	0.14	1.59	0.12
BMI	0.13	1.35	0.18
Mean IMT	0.10	1.14	0.26

Objective variables: components of metabolic syndrome (0, 1, 2, and 3). *n* = 76, *R* = 0.71, *P* < 0.0001. HOMA-IR: homeostasis model assessment of insulin resistance; MBR: mean blur rate; BOS: blowout score; BMI: body mass index; IMT: intima-media thickness.

**Table 6:** Results of a single-regression analysis and multiple-regression analysis for factors independently contributing to BOS-Choroid.

Explanatory variables	Single regression		Multiple regression		
	*r*	*P*	Standard regression	*t* value	*P*
Age	−0.45	<0.0001	−0.41	−3.47	0.0009
Men = 1, women = 0	0.16	0.17			
BMI	−0.05	0.67			
Spherical refraction	−0.13	0.27			
IOP	0.04	0.77			
OPP	−0.001	0.99			
Heart rate	0.28	0.01	0.44	4.89	<0.0001
Mean IMT	−0.01	0.95			
e' velocity	0.28	0.01	0.02	0.15	0.88
E/e' ratio	−0.24	0.04	−0.19	−1.83	0.07
Total cholesterol	−0.03	0.78			
LDL-C	0.03	0.80			
Hematocrit	0.30	0.009	0.15	1.46	0.15
Creatinine	0.06	0.62			
MetS components (0, 1, 2, and 3)	−0.27	0.02	−0.31	−3.41	0.001

Objective variables: BOS-Choroid; *r* = 0.70, *P* < 0.0001. *n* = 76. BOS: blowout score; BMI: body mass index; IOP: intraocular pressure; OPP: ocular perfusion pressure; IMT: intima-media thickness; LDL-C: low-density lipoprotein cholesterol; HOMA-IR: homeostasis model assessment of insulin resistance; MetS: metabolic syndrome. The correlation coefficient did not show more than 0.6 among the all explanatory variables.

**Table 7:** Results of a single-regression analysis and multiple-regression analysis for factors independently contributing to MBR-Tissue (temporal).

Explanatory variables	Single regression		Multiple regression		
	*r*	*P*	Standard regression	*t* value	*P*
Age	−0.10	0.39			
Men = 1, women = 0	−0.07	0.53			
BMI	−0.17	0.15			
Spherical refraction	0.01	0.90			
IOP	0.06	0.59			
OPP	−0.14	0.22			
Heart rate	−0.25	0.03	−0.20	−1.87	0.07
Mean IMT	−0.28	0.01	−0.22	−2.03	0.046
e' velocity	0.11	0.33			
E/e' ratio	−0.17	0.14			
Total cholesterol	0.01	0.96			
LDL-C	−0.01	0.92			
HOMA-IR	−0.08	0.48			
Hematocrit	−0.05	0.70			
Creatinine	0.14	0.22			
MetS components (0, 1, 2, and 3)	−0.40	0.0004	−0.31	−2.84	0.006

Objective variables: MBR-tissue (temporal); *r* = 0.48, *P* = 0.0002. *n* = 76. MBR: mean blur rate: blowout score; BMI: body mass index; IOP: intraocular pressure; OPP: ocular perfusion pressure; IMT: intima-media thickness; LDL-C: low-density lipoprotein cholesterol; HOMA-IR: homeostasis model assessment of insulin resistance; MetS: metabolic syndrome. The correlation coefficient did not show more than 0.6 among the all explanatory variables.
